# Blood Vessel Formation and Bone Regeneration Potential of the Stromal Vascular Fraction Seeded on a Calcium Phosphate Scaffold in the Human Maxillary Sinus Floor Elevation Model

**DOI:** 10.3390/ma11010161

**Published:** 2018-01-20

**Authors:** Elisabet Farré-Guasch, Nathalie Bravenboer, Marco N. Helder, Engelbert A. J. M. Schulten, Christiaan M. ten Bruggenkate, Jenneke Klein-Nulend

**Affiliations:** 1 Department of Oral Cell Biology, Academic Centre for Dentistry Amsterdam (ACTA), University of Amsterdam and Vrije Universiteit Amsterdam, Amsterdam Movement Sciences, Amsterdam 1081 LA, The Netherlands; e.farreguasch@acta.nl; 2 Department of Oral and Maxillofacial Surgery, VU University Medical Center/Academic Centre for Dentistry Amsterdam (ACTA), Amsterdam Movement Sciences, Amsterdam 1081 HV, The Netherlands; m.helder@vumc.nl (M.N.H.); eajm.schulten@vumc.nl (E.A.J.M.S.); cmtenbruggenkate@alrijne.nl (C.M.t.B.); 3 Department of Clinical Chemistry, VU University Medical Center, Amsterdam Movement Sciences, Amsterdam 1007 MB, The Netherlands; n.bravenboer@vumc.nl

**Keywords:** angiogenesis, bone, adipose stem cells, calcium phosphates, bone substitutes, clinical translation, clinical trials, adipose

## Abstract

Bone substitutes are used as alternatives for autologous bone grafts in patients undergoing maxillary sinus floor elevation (MSFE) for dental implant placement. However, bone substitutes lack osteoinductive and angiogenic potential. Addition of adipose stem cells (ASCs) may stimulate osteogenesis and osteoinduction, as well as angiogenesis. We aimed to evaluate the vascularization in relation to bone formation potential of the ASC-containing stromal vascular fraction (SVF) of adipose tissue, seeded on two types of calcium phosphate carriers, within the human MSFE model, in a phase I study. Autologous SVF was obtained from ten patients and seeded on β-tricalcium phosphate (*n* = 5) or biphasic calcium phosphate carriers (*n* = 5), and used for MSFE in a one-step surgical procedure. After six months, biopsies were obtained during dental implant placement, and the quantification of the number of blood vessels was performed using histomorphometric analysis and immunohistochemical stainings for blood vessel markers, i.e., CD34 and alpha-smooth muscle actin. Bone percentages seemed to correlate with blood vessel formation and were higher in study versus control biopsies in the cranial area, in particular in β-tricalcium phosphate-treated patients. This study shows the safety, feasibility, and efficiency of the use of ASCs in the human MSFE, and indicates a pro-angiogenic effect of SVF.

## 1. Introduction

Retention of removable dentures is often a problem for edentulous patients with severe maxillary or mandibular atrophy, a common problem in the aged population. Installation of dental implants can help these patients by providing better support for and retention of their dental prosthesis. Placing dental implants requires sufficient jaw bone volume, which is often not available, especially in the edentulous distal maxillary area of severely atrophied maxillae. In these cases, the bone volume must be increased by augmentation prior to dental implant placement.

Maxillary sinus floor elevation (MSFE) or augmentation has been introduced to dentistry in the mid-1970s (modified by Tatum in 1986) [1] and has become part of pre-prosthetic surgery. In this surgical procedure, the distal area of the maxilla is augmented by transplanting bone or bone substitutes to the bottom of the maxillary sinus. The maxillary sinus floor elevation model is unique by allowing histological examination of biopsies obtained during the preparation for dental implant placement using a hollow trephine bur.

In dentistry, autologous bone grafting is still the “gold standard” for bone augmentation. Autografting, however, has an important disadvantage, i.e., trabecular bone needs to be harvested from elsewhere in the skeleton, usually from the lateral mandible, the chin, or the iliac crest, and applied to the jaw defect. This means another operation site, including the risk of hospitalization, causing donor site morbidity, and potential complications, such as post-operative infections, and functional defects [2]. Therefore, alternative bone substitute materials have been evaluated. Calcium phosphates, such as hydroxyapatite (HA), β-tricalcium phosphate (β-TCP), and a combination of HA/β-TCP, are often used since they do not evoke adverse cellular reactions and, in time, the material is either replaced by bone or integrated into the body, depending on the degradation properties [3,4,5]. However, these materials have limitations due to the slow speed of osteoconduction when compared to the properties of autologous bone [6,7,8]. The time required is strongly determined by the time that osteogenic cells take to grow into the augmentation space and would potentially be shortened if bone regeneration-competent cells (mesenchymal stem cells; MSCs) are mixed with the bone substitute. The calcium phosphate carrier has controlled 3D properties allowing immediate colonization by MSCs of the entire volume of the ceramics, and in depth revascularization [9,10]. 

Endothelial cells are lining blood vessels and allow the formation of new blood capillaries by the sprouting of an existing small vessel, a phenomenon called angiogenesis. This process allows tissue growth and repair by extending and remodeling the network of blood vessels [11,12]. In contrast to small blood vessels, composed of endothelial cells surrounded by a basal lamina and loosely covered by single pericytes, larger vessels are coated with multiple layers of smooth-muscle cells and elastic and collagenous fibers [6]. These are composed by the endothelium, a thin layer of endothelial cells, separated from the surrounding outer layers by a basal lamina. The amounts of connective tissue and smooth muscle in the vessel wall vary according to the vessel’s diameter and function, but the endothelial lining is always present. Pericytes have been associated mainly with stabilization and hemodynamic processes of blood vessels. Their functions are, however, much more diverse than traditionally thought. They can sense angiogenic stimuli, guide sprouting tubes, elicit endothelial survival functions, and even exhibit macrophage-like activities which make them crucial cells in the process of tissue repair and remodeling [13].

Poor angiogenesis is a common and vital barrier to tissue regeneration. Regenerating tissue over 200 µm exceeds the capacity of nutrient supply and waste removal from the tissue and, therefore, requires an intimate supply of vascular networks [14]. This has led to the use of angiogenic growth factors and/or transplantation of proangiogenic cells, such as endothelial progenitor cells (EPCs), in combination with scaffolds. The use of angiogenic growth factors and/or transplantation of these proangiogenic cells only, however, also has disadvantages, since perivascular cells, including mural cells, are obligatory for the formation of native, multilayered mature microvessels [15]. The potential of MSCs to stimulate angiogenesis holds interesting promises to the field of tissue engineering.

Adipose tissue may be easily obtained from patients using liposuction or oral surgical procedures [16]. Adipose tissue represents a promising source of MSCs, as liposuction can be performed with minimal patient discomfort and yields higher numbers of MSCs than bone marrow, which could avoid costly cell expansion to obtain a number of cells high enough for clinical use [17,18,19]. Risks associated with cell culturing such as pathogen contamination, spontaneous transformation and loss of proliferation and differentiation potential are thus minimized [20,21,22]. The stromal vascular fraction (SVF), obtained immediately after digestion of adipose tissue with collagenase and centrifugation to separate the floating adipocytes, is highly heterogeneous and contains many cell subsets, including native adipose stem cells (ASCs), mature endothelial cells, and haematopoietic cells [19]. SVF also contains macrophages, which secrete a multitude of vascular growth factors and cytokines [23]. The ASCs in SVF have been shown to attach, proliferate, and osteogenically differentiate on calcium phosphate scaffolds [24], and secrete a high number of growth factors [25]. ASCs have not only shown osteogenic potential in vivo [26,27], but also demonstrated angiogenic potential crucial for bone tissue engineering applications in mice [28]. This supports in vitro observations that ASCs in SVF secrete a variety of angiogenic and anti-apoptotic growth factors [29], and that SVF is highly enriched with CD34+CD45− cells. The CD34+ cells are capable of stimulating angiogenesis, and are involved in neovascularization processes that facilitate healing of ischemic tissues in mouse models [30]. However, whether SVF/ASCs are also effective in stimulating vascularization in humans has not been unequivocally shown so far. 

In an earlier performed clinical phase I study [27], employing our one-step surgical procedure [18] in patients undergoing maxillary sinus floor elevation, we isolated SVF, and re-implanted them intraoperatively into the patient again. This study successfully showed feasibility, safety, and potential efficacy of using bioactive implants consisting of calcium phosphate carriers seeded with freshly isolated SVF containing ASCs. Since we hypothesized that the SVF will positively contribute not only to bone formation, but likely also to vascularization, the current study aimed to evaluate vascularization in relation to bone formation potential of SVF in biopsies from the previous study obtained after six months (when dental implant placement occurred). For this evaluation, a recently developed immunohistochemical staining technique for methyl methacrylate (MMA) polymer resin embedded bone biopsies [31] was applied.

## 2. Materials and Methods

### 2.1. Clinical Study Outline

This angiogenic study is an extension of a phase I/IIa clinical trial study we reported on before [27], in which safety and potential efficacy of maxillary sinus floor bone augmentation using calcium phosphate bone substitutes and a freshly-isolated adipose stem cell preparation, termed the stromal vascular fraction (SVF), was evaluated in a one-step surgical procedure in 10 partially-edentulous patients requiring dental implants for prosthetic rehabilitation. The clinical study was registered in the Netherlands Trial Registry (NTR4408), and complied with the principles of the Declaration of Helsinki of 1975, revised in 2008. All protocols were approved by the medical ethics committee (IRB) of the VU University Medical Center Amsterdam, as well as the central committee on research involving human subjects (CCMO, The Hague, The Netherlands; Dossier number: NL29581.000.09; EudraCT-number: 2009-015562-62). All patients signed a written informed consent before participation in the study. For detailed inclusion and exclusion criteria, procedures and assessments according to the study protocol of our previously performed phase I trial, one is referred to our previous report [27]. The demographic data of our patient group is summarized in Table 1.

**Figure 1:**
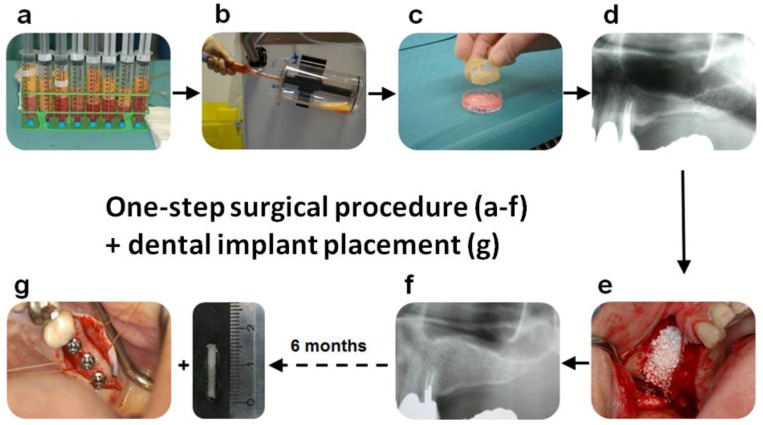
Concept of a sinus floor elevation with freshly isolated adipose-derived stem cells in a one-step surgical procedure. (**a**) The adipose tissue and liposuction fluid obtained by liposuction is collected in syringes; (**b**) the filled syringes are transferred into a Celution 800/CRS system to obtain the fresh stromal vascular fraction containing the adipose stem cells; and (**c**) the freshly isolated adipose stem cells are seeded onto the calcium phosphate scaffold. Unattached cells are washed off. During the short attachment period of the cells (30 min), the patient is prepared for the maxillary sinus floor elevation procedure via a lateral approach; (**d**) the anatomical selection criterion was a pre-existing (native) alveolar bone height of >4 mm obtained from the preoperative panoramic radiograph; and (**e**) after reflection of the mucoperiosteal flap, a bony window is created in the lateral wall of the maxillary sinus and carefully moved and rotated medially toward the maxillary sinus, after dissection of the maxillary sinus mucosa (trap-door technique). The calcium phosphate scaffold is inserted immediately into the patient, and the space created is filled with the bone substitute combined with the adipose stem cells. Finally, the wound is closed; (**f**) after a healing period of five months post-MSFE (prior to dental implant placement), a panoramic radiograph is made to determine the increase in vertical height bone + bone substitute at the planned dental implant positions; and (**g**) after six months, bone biopsies are taken by using a hollow burr for histomorphometrical and immunohistochemical analysis, and dental implants are placed.

A graphic visualization of the surgical protocol is depicted in Figure 1. Briefly, the surgery started by collecting >125 mL of adipose tissue using a syringe-based lipoaspiration. The lipoaspirate (Figure 1a) was subsequently transported to a special stem cell laboratory within the VU University Medical Center Amsterdam operation complex. Within the stem cell laboratory, the adipose tissue was processed with the CE marked Celution device (Cytori Therapeutics, Inc., San Diego, CA, USA) (Figure 1b) to obtain SVF. Viability and cell number was determined in triplicate with a Nucleocounter NC-100 (ChemoMetec A/S, Allerød, Denmark) according to the manufacturer’s protocol. The release criterion was set at ≥70% viability. For implantation cells were seeded in Ringer’s lactate solution in a concentration of 10^7^ nucleated SVF cells (±2 × 10^5^ ASC-like cells)/g calcium phosphate carrier (Figure 1c). Calcium phosphate carriers consisted of 100% Ceros^®^ β-TCP with 60% porosity and granule size of 0.7–1.4 mm (Thommen Medical, Grenchen, Switzerland) or Straumann^®^Bone Ceramic biphasic calcium phosphate (BCP), consisting of 60% hydroxyapatite (HA) and 40% β-tricalcium phosphate (β-TCP) with 90% porosity and granule size of 0.5–1.0 mm (Straumann AG, Basel, Switzerland). After allowing attachment of the cells for 30 min and subsequent washing with Ringer’s lactate solution to remove unattached cells, the carriers were implanted using a standard maxillary sinus floor elevation procedure according to the lateral “top hinge trap door” procedure of Tatum [1] (Figure 1d–f). In the case of a bilateral “split-mouth” design treatment, one side was implanted with the cell-seeded carrier, while the other (control) side was implanted with carriers undergoing the same seeding procedure, but with vehicle (Ringer’s lactate solution) only.

Bone biopsies were obtained during dental implant surgery after a six month healing period, using hollow trephine drills with an external diameter of 3.5 mm (Straumann AG, Basel, Switzerland) under local anesthesia prior to dental implant placements (Figure 1g). After a three month osseointegration period the suprastructures were manufactured, and placed by the patient’s dentist.

### 2.2. Biopsy Processing and Evaluation

**Figure 2:**
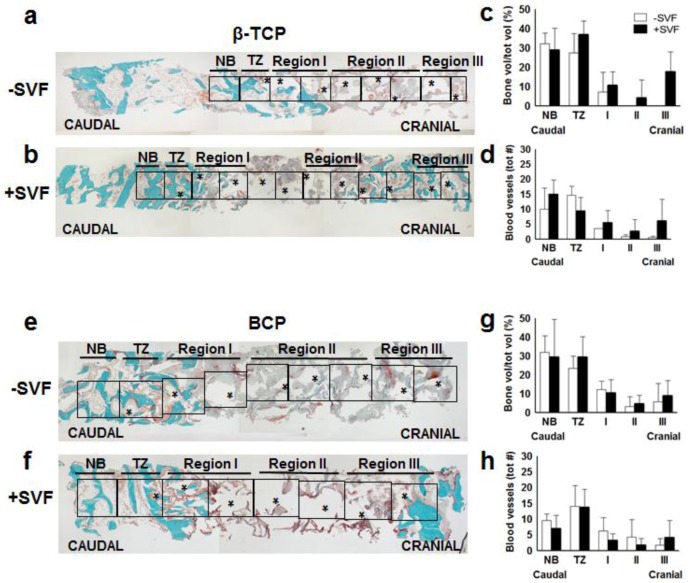
Histomorphometrical analysis of maxillary bone biopsies from patients treated with β- TCP (**a**–**d**) and BCP (**e**–**h**). Graphs a and b on the one side, and e and f on the other side represent typical bilateral biopsies from one and the same patient, while graphs c, d, g, and H represent quantifications of 3–5 samples. Midsagittal histological sections of each biopsy were stained with Goldner’s Trichome method [3], to distinct mineralized bone tissue (green) and unmineralized osteoid (red). Biopsies were divided in consecutive 1 mm^2^ regions of interest (ROIs). The transition zone (TZ) indicates the first ROI where graft material (*) was observed when analyzing from the caudal to the cranial side of the biopsy. Bone ingrowth is determined from the sinus floor towards the cranial side of the biopsies. Original magnification is 100×. Since the biopsies had different lengths we decided to define them in three regions after the transition zone. The ROI of the native bone (NB) next to the TZ was also analyzed. Percentage mineralized bone vol/tot vol from control sides without stem cells (white bars; *n* = 3), and study sides with stem cells (black bars; *n* = 5) from patients treated with β-TCP (**b**) or BCP (**f**) are depicted. Total number of blood vessels from control sides without stem cells (white bars; *n* = 3), and study sides with stem cells (black bars; *n* = 5) from patients treated with β-TCP (**d**) or BCP (**h**). β-tricalcium phosphate; BCP, biphasic calcium phosphate; NB, native bone; TZ, transition zone; SVF, stromal vascular fraction; bone vol/tot vol, bone volume/total volume; tot #, total number.

The biopsies were fixed in 4% formaldehyde solution (Klinipath BV, Duiven, The Netherlands) at 4°C for 24 h, removed from the drill, transferred to 70% ethanol, and stored until use for histomorphometrical analysis (Figure 2), as described below. For a valid, uniform comparison of the biopsies taken from the sides that were treated with calcium phosphate only and the biopsies taken from the sides augmented with calcium phosphate and ASCs, a selection was made by two independent experienced observers. The biopsies taken from implant sides outside the augmented maxillary sinus (mainly implant position 14 and/or 24) were excluded from analysis. Per patient, one biopsy from each side was selected in the middle of the grafted area to exclude the effect of surrounding bone containing mechanically-loaded dental elements and bone near the nasal wall of the maxillary sinus. Using these selection criteria, a total of 16 selected biopsies (six from the control side, 10 from the study side) from 10 patients were studied to analyze the blood vessel formation and bone formation (Table 1, bold numbers). In addition, one sample from a transilical biopsy was used as control for the immunohistochemical analysis.

### 2.3. Histology and Histomorphometry

After dehydration in descending alcohol series, the bone specimens were embedded without prior decalcification in low temperature polymerizing methylmethacrylate (MMA, Merck Schuchardt OHG, Hohenbrunn, Germany). Longitudinal sections of 5 μm thickness were prepared using a Jung K microtome (R. Jung, Heidelberg, Germany). Midsagittal histological sections of each biopsy were stained with Goldner’s Trichome method [6], in order to distinguish mineralized bone tissue (green) and unmineralized osteoid (red). The histological sections were divided into regions of interest (ROI) of 1 mm^2^ for blinded histomorphometrical analysis, as previously described [32]. Depending on the length of the biopsy, the number of ROIs ranged from 9–15. The digital images of the scanned biopsies were analyzed, starting from the caudal side of the biopsy, and continuing towards the cranial side. This method allowed to compare similar ROIs for all biopsies (with and without stem cells) with respect to the bone regeneration and blood vessel formation in the augmented maxillary sinus. For each ROI, the bone volume (BV) was calculated as a percentage of the total tissue volume (TV), as previously described [33]. This analysis was performed by two independent blinded observations.

For each separate area of interest, the histomorphometrical measurements were performed with a computer using an electronic stage table and a Leica DC 200 digital camera (Leica, Wetzlar, Germany). The computer software used was Leica QWin© (Leica Microsystems Image Solutions, Rijswijk, The Netherlands). Digital images of the sections were acquired at 100× magnification. Consecutive ROI of 1 mm^2^ each were defined and numbered throughout the whole biopsy. The transition zone (TZ) indicates the first ROI where graft material was observed when analyzing from the caudal to the cranial side of the biopsy. Because the biopsies analyzed had different lengths, we decided to define them in three regions after the transition zone (TZ) between the native bone or caudal area and the scaffold area towards cranial. The first two ROIs on the right of the transition zone were defined as region I, the two or three ROIs in the middle (even or odd numbers) as region II, and the two rightmost ROIs as region III (Figure 2).

Data from the residual native bone part of the biopsy next to the transition zone and for each area from the sinus floor towards the cranial side of the biopsy was analyzed separately (Figure 2). Similar to this method we have already been able to compare similar areas of interest for the two sides (control and test side) with respect to the bone regeneration performance indicated by the amount of osteoid and bone formed, and volume of remaining graft material [32].

Blood vessel numbers, taking into account the blood vessel size, were determined as mean value of two separate blinded counts. Blood vessel size was calculated as the total blood vessel area expressed in µm^2^. According to their size, blood vessels were divided into small (0–400 µm^2^) or large vessels (>400 µm^2^).

### 2.4. Immunohistochemistry

To quantify microvessel density, immunohistochemical staining for CD34, a marker of endothelial cells, as well as stem cells, such as endothelial progenitor stem cells and hematopoietic stem cells, was performed [34,35]. The expression of smooth muscle actin (SMA), a marker of smooth muscle cells as well as pericytes, was also analyzed by immunohistochemistry [35,36]. The presence of pericytes and smooth muscle cells surrounding blood vessels has been described as a structural parameter indicative of vascular maturity. 

The MMA in the sections was removed by immersing them in xylene/chloroform (Merck, Darmstadt, Germany) for 30 min at room temperature followed by two rinses with xylene. Bone sections were rehydrated in graded alcohol solutions. To block endogenous peroxidase when using the avidin biotin peroxidase complex the hydrated specimens were transferred to 3% (*v*/*v*) hydrogen peroxide in methanol for 15 min. In a set of pilot experiments, it was deduced that optimal CD34 staining results were achieved with 5 µg/mL proteinase K pretreatment for 10 min (Invitrogen, Carlsbad, CA, USA), while SMA detection was best without proteinase K predigestion. Non-specific binding of immunoglobulin G was blocked by incubation with 5% (*v*/*v*) normal serum (as appropriate for each antibody) for 60 min with 0.1% bovine serum albumin (Sigma, St Louis, MI, USA). The sections were then incubated with the primary antibodies at optimal dilutions for 2 h at room temperature (SMA Monoclonal Mouse Anti-Human Smooth Muscle Actin Clone 1A4 1:50, Dako, Carpinteria, MI, USA) and overnight incubation at 4 °C in a humidified chamber (CD34 Monoclonal Mouse Anti-Human CD34 Class II Clone QBEnd-10 1:20, Dako). Sections were rinsed in phosphate-buffered saline and incubated at room temperature with an horseradish peroxidase (HRP)-labelled polymer conjugated with secondary antibody (Envision Kit, Dako, Santa Clara, CA, USA), for 0.5 h at room temperature before detection using aminoethyl carbazole (AEC) (Invitrogen, Carlsbad, CA, USA) staining as recommended by the manufacturer’s protocol. Sections were counterstained with haematoxylin (Merck, Schuchardt OHG, Hohenbrunn, Germany). Sclerostin (hSOS, Dako, Santa Clara, CA, USA) was used as positive control. Negative controls (without primary or secondary antibodies, or both) were also performed for all antibodies tested.

The determination of the number of blood vessels was performed as previously described [37]. The number of blood vessels was expressed per area of soft connective tissue (mm^2^) [37]. Blood vessel size was calculated as described above. 

### 2.5. Statistics

Data are presented as mean ± standard deviation (SD). Data analysis and statistical analysis were performed using GraphPad Prism 5 software (GraphPad Software, La Jolla, CA, USA) and IBM SPSS 23 statistical software (CircleCI, San Francisco, CA, USA). Selected biopsies from all treated patients (with vs. without stem cells) were compared between the β-TCP and BCP groups.

A paired Wilcoxon signed rank test was performed to assess whether bone volume and blood vessel number were higher at the study sides compared to control sides for each material. An unpaired nonparametric Mann Whitney U test was performed to test differences between β-TCP and BCP in control and study samples. Statistical significance was considered if *p*-values were <0.05.

## 3. Results

### 3.1. Clinical Evaluation and Implant Survival

Patient data and selected biopsies are listed in Table 1. As control for the immunohistochemical analysis, transiliacal bone biopsies and paraffin-embedded maxillary bone biopsies were used for the assays. 

### 3.2. Data Analysis

The biopsies analyzed were obtained from a clinical phase I study in 10 patients, which was primarily aimed at assessing feasibility and safety of a one-step surgical concept applying the freshly isolated SVF containing adipose stem-cell like cells in combination with calcium phosphate bone substitutes. We are aware that our sample size is relatively low and, therefore, we mainly focused on the patterns obtained in this results section. Nevertheless, we felt that it is relevant to include the statistical analyses performed as additional information for the reader to judge our findings, but would like to state that our statistical analyses as described below should be seen as indications and not as solid proof for our statements.

### 3.3. Quantitative Histomorphometric Evaluation

All bone biopsies were analyzed from caudal (native bone) to cranial (scaffold area). In contrast to the previous report [27], we now divided the graft area in three distinct regions, and analyzed angiogenesis in the biopsies. 

In the vehicle-treated (control) β-TCP group, we observed that the mineralized bone volume decreased from the residual native bone next to the transition zone towards the cranial side of the biopsies, and that no bone formation was present in the most cranial area, designated as region III (Figure 2a). In confirmation with the previous study, when analyzing the bone biopsies in the SVF-treated β-TCP group, higher bone volumes per total volume were found in region III in 80% (four out of five) of the patients (Figure 2b). When comparing the biopsies from the SVF-supplemented sides with the control sides, a marked although non-significant difference in the bone volumes in region III (SVF-treated vs. control: 17.7 ± 10.3% vs. 0 ± 0%) (*p* = 0.1) was observed (Figure 2c). When analyzing blood vessel formation in a similar way, a higher blood vessel number was found in all regions of the scaffold area in the SVF-treated group vs. the control group (Figure 2d), but these differences did not reach significance.

In the BCP group, the mineralized bone volume decreased towards the cranial side of the biopsies. In 33% (one out of three) of the control sides, bone formation in region III was observed (Figure 2e), whereas this occurred in 60% (three out of five) of the SVF-supplemented sides (Figure 2f). Moreover, a higher percentage of bone volume per total volume was found in SVF-treated vs. control sides in this region (BCP: 8.8 ± 8% vs. 5.6 ± 9.8%), although this was less prominent compared to the findings in the β-TCP treatments (Figure 2g). This was also the case for the blood vessel formation in region III (Figure 2h).

Comparison of the bone contents between the control and SVF-supplemented group per biomaterial (Figure 2c,g) in each region showed no significant differences. When comparing the two biomaterials for their effect on bone formation in the presence or absence of SVF, it could be concluded that β-TCP, but not BCP, displayed increased bone formation in combination with SVF, but only in region III (*p* = 0.047). In the absence of SVF, no difference between the two biomaterials was observed.

When analyzing the relative percentages of small and large blood vessels in the different regions of the scaffold area, we observed that the control sides in the β-TCP group showed a similar percentage of small and large blood vessels (~70%) in region I which both decreased gradually from caudal towards cranial to ~10% in region III (Table 2). Interestingly, the SVF-supplemented group showed a different pattern: instead of a steady decrease, a biphasic pattern could be observed, i.e., high vessel percentages (~40% with an approximate 1:1 ratio between small and large vessels) in region I, four-fold lower percentages in region II for both vessel sizes, and another ~40% and 1:1 ratio in region III. 

The control sides of the BCP group displayed a similar pattern as described for the control β-TCP sides, with a gradual decrease of blood vessel formation from region I to region III, although region I showed a slightly different percent ratio (65:45) between large and small vessels, which leveled to ~15% each at a 1:1 ratio (Table 2). Again, different patterns were observed in the SVF-supplemented BCP group, i.e., for the small vessels, region I and III showed equal percentages and even an increase in region II, while the large vessel counts showed the highest number in region I, low numbers in region II, and intermediate numbers in region III. Please refer to Supplementary Materials Table S1 for more detailed information on the percentages of small and large blood vessels from maxillary bone biopsies obtained from patients treated with β-TCP and BCP without stem cells and with stem cells, as analyzed by histomorphometrical and immunohistochemical analyses.

### 3.4. Immunohistochemistry

We found that tissue processing techniques preserved original morphology, while scratch artifacts, folds, and distensions were absent in all sections.

#### 3.4.1. CD34+ Blood Vessels

**Figure 3:**
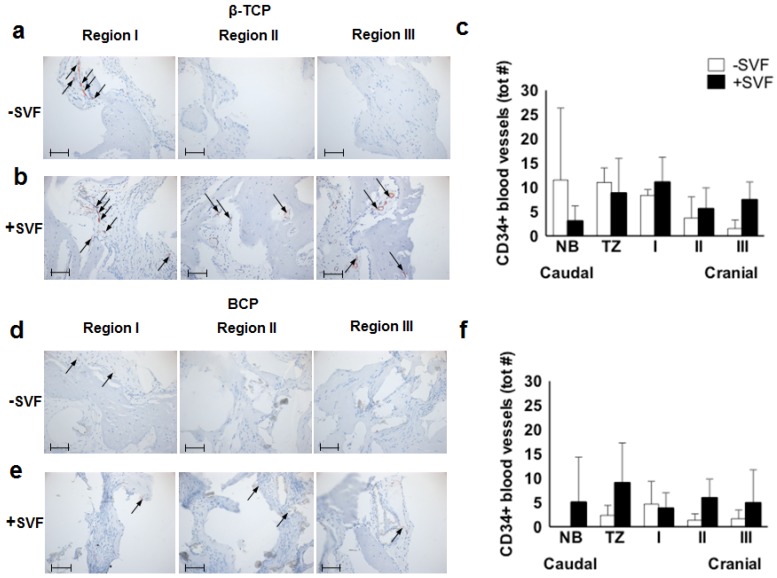
Immunohistochemical analysis of CD34, a marker of endothelial cells as well as stem cells such as endothelial progenitor stem cells and hematopoietic stem cells, of a maxillary bone biopsy from a patient treated with β-TCP (**a**–**c**) and BCP (**d**–**f**). Magnification: 200×. The scale bar represents 100 μm. The total number of CD34+ blood vessels of selected bone biopsies taken from control sides without stem cells (white bars; *n* = 3), and study sides with stem cells (black bars; *n* = 4) from patients treated with β-TCP (**b**) or BCP (**e**). β-TCP, β-tricalcium phosphate; BCP, biphasic calcium phosphate; NB, native bone; TZ, transition zone; SVF, stromal vascular fraction; tot #, total number.

We observed CD34 staining around vessels in the maxillary bone samples of methyl-methacrylate embedded tissue of patients augmented with β-TCP or BCP (Figure 3a–f). 

In the control sides of the β-TCP group, the total number of CD34+ blood vessels decreased from region I to III, resulting in only scarce expression in region III (Figure 3a). More staining, particularly in regions II and III, was observed in the SVF-treated sides of the β-TCP group (Figure 3b). When quantifying the stained vessels in the β-TCP group, all three graft regions showed higher numbers of CD34+ vessels in the SVF-supplemented vs. the control sides with the largest difference in region III, but none reached statistical significance (Figure 3c).

In the BCP group, both the control (Figure 3d) and the SVF-supplemented sides (Figure 3e) showed similar patterns as described for the β-TCP group. Quantification indicated stable total CD34+ vessel numbers for the SVF-seeded scaffolds which were higher in region II and in region III when compared to the control sides (Figure 3f).

When analyzing the relative percentages of CD34+ small and large blood vessels in the different regions of the scaffold area (Table 2), we observed that in the control sides of the β-TCP group a similar percentage of small and large blood vessels was found in region I (~70% of the total CD34+ vessels in the grafted area), which decreased gradually from caudal towards cranial. In the stem cell-treated β-TCP group a comparable pattern was found except for region III, where the percentage of large blood vessels increased again to even higher values than in region I (from 40% to 60%).

In the control sides of the BCP group, the percentage of small vessels declined as described for their counterparts in the β-TCP group, but the number of large vessels again increased from region II to region III. In the SVF-supplemented augmentations comparable patterns were observed, but in region II both the small and large vessels were considerably higher, indicating more advanced angiogenesis in the core of the grafted material (Table 2). 

#### 3.4.2. SMA+ Blood Vessels

**Figure 4:**
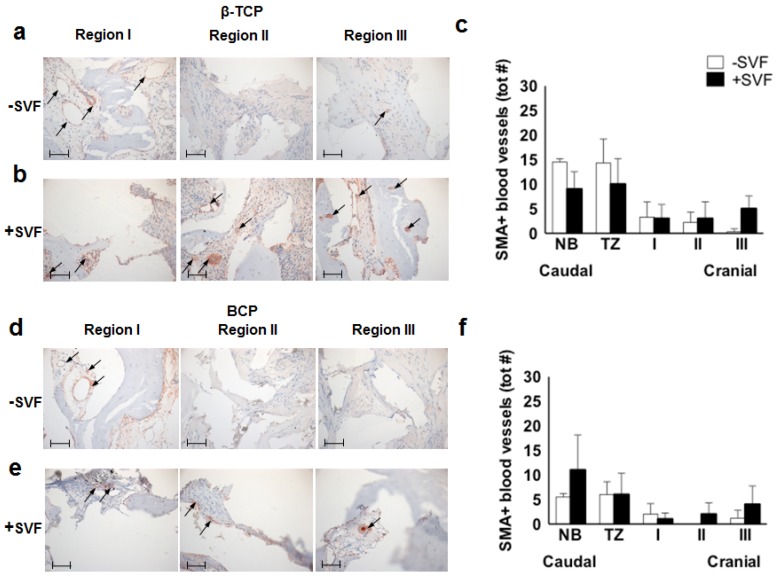
Immunohistochemical analysis of α-smooth muscle actin (SMA), a marker of pericytes, as well as smooth muscle cells present in the blood vessel walls, of a maxillary bone biopsy from a patient treated with β-TCP (**a**–**c**) and BCP (**d**–**f**). Magnification: 200×. The scale bar represents 100 μm. The total number of SMA+ blood vessels of selected bone biopsies taken from control sides without stem cells (white bars; *n* = 3), and study sides with stem cells (black bars; *n* = 5) from patients treated with β-TCP (**b**) or BCP (**e**). β-TCP, β-tricalcium phosphate; BCP, biphasic calcium phosphate; NB, native bone; TZ, transition zone; SVF, stromal vascular fraction; tot #, total number.

Immunostaining for SMA, indicative for more mature blood vessels, resulted in relatively low numbers of SMA+ blood vessels in the grafted area. When comparing the control (Figure 4a) and SVF-supplemented (Figure 4b) sides of the β-TCP group, the number of SMA+ vessels was low for both treatments in regions I and II. Quantification showed differential numbers of SMA+ stained vessels in region III: no SMA+ vessels in the control sides, while slightly increased SMA+ blood vessel numbers (relative to regions I and II) in the SVF-supplemented sides (Figure 4c). Similar patterns were observed in the BCP group (Figure 4d–f). 

When analyzing the relative percentages of SMA+ small and large blood vessels in the different regions of the scaffold area (Table 2), we observed in the control sides of the β-TCP group equal levels of small and large vessels in regions I and II, and a sharp drop of both types of vessels in region III. In the SVF-supplemented sides of this group, a similar but lower relative percentage was found, but now with a 2–3 fold increase in region III. In the control sides of the BCP group, blood vessels were only observed in regions I and III, in a 3–4:1 ratio. In contrast, the SVF-supplemented sides of the BCP group showed a divergent pattern, i.e., in regions I and II equal levels of small and large vessels were seen, but in region III a strong increase in large vessels and concomitant decrease in smaller vessels was observed, indicating more mature vessel formation in region III upon SVF supplementation.

**Figure 5:**
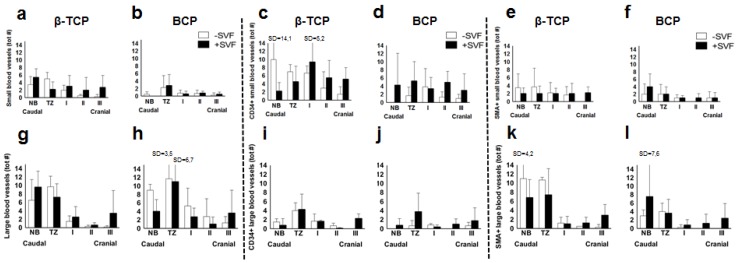
Histomorphometrical analysis of the absolute number of small and large blood vessels from maxillary bone biopsies in patients treated with β-TCP (**a**,**g**) and BCP (**b**,**h**) with stem cells (black bars, *n* = 5) and without stem cells (white bars, *n* = 3). The total number of CD34+ small and large blood vessels from maxillary bone biopsies in patients treated with β-TCP (**c**,**i**) and BCP (**d**,**j**) with stem cells (black bars, *n* = 5) and without stem cells (white bars, *n* = 3). The total number of SMA+ small and large blood vessels from maxillary bone biopsies in patients treated with β-TCP (**e**,**k**) and BCP (**f**,**l**) with stem cells (black bars, *n* = 5) and without stem cells (white bars, *n* = 3). See the legend to Figure 2 for the definitions of regions I-III. β-TCP, β-tricalcium phosphate; BCP, biphasic calcium phosphate; NB, Native bone; TZ, transition zone; SVF, stromal vascular fraction; tot #, total number.

When analyzing the total blood vessel numbers (Figure 2d,h) and the subdivision in small and large vessels (Figure 5a,b,g,h) of the control and SVF-supplemented sides per biomaterial in each region, no significant differences were observed.

A comparison of the CD34+ blood vessel total numbers (Figure 3c,f) and the subdivision in small and large vessels (Figure 5c,d,i,j) of the control and SVF-supplemented sides per biomaterial in each region showed no significant differences. When comparing the two biomaterials for their effect on CD34+ vasculogenesis in the presence or absence of SVF, it could be concluded that SVF supplementation significantly promoted CD34+ large vessel formation in region I in β-TCP, but not BCP (*p* = 0.017). In the absence of SVF, no difference between the two biomaterials was observed.

Comparison of the SMA+ blood vessel total numbers (Figure 4c,f) and the subdivision in small and large vessels (Figure 5e,f,k,l) of the control and SVF-supplemented sides per biomaterial in each region showed no significant differences. When analyzing the two biomaterials for their effect on SMA+ vasculogenesis in the presence or absence of SVF, it could be concluded that β-TCP in the absence of SVF significantly promoted total SMA+ vessel numbers (*p* = 0.037) and, in particular, large SMA+ vessels (*p* = 0.025) in region II when compared to BCP. In the presence of SVF, no difference between the two biomaterials was observed.

## 4. Discussion

In bone tissue engineering adequate vascularization is crucial for timely and adequate transport of nutrients and waste removal, and the provision of progenitor cells for tissue remodeling and repair. It is widely agreed that vascularization and bone formation are highly linked, and that vascularization precedes osteogenesis during both embryonic development and adult bone healing [38]. Since we hypothesized that supplementation of bone substitutes with SVF, containing adipose stem cells, will positively contribute not only to bone formation, as shown previously [27], but likely also to vascularization, the current study aimed to evaluate vascularization (number and size of blood vessels) in relation to bone formation potential of SVF.

Histomorphometric analysis and quantitative assessments of CD34- and SMA-immunostained blood vessels showed: (i) a clear trend towards increased bone formation in the SVF-supplemented group vs. the control group, in particular in the most cranial part (region III) of the biopsies, which was significantly higher in the β-TCP vs. the BCP group; (ii) comparable patterns of angiogenic and bone formation levels; (iii) comparable total blood vessel numbers obtained from histomorphometric and immunohistochemical analyses; (iv) SVF significantly promoted CD34+ large vessel formation in region I in β-TCP, but not BCP; (v) in the BCP group supplemented with SVF, region II displayed considerably higher CD34+ small and large vessel numbers; and (vi) in the absence of SVF the β-TCP, but not the BCP scaffolds, promoted both small and large vessel numbers in region II.

Our data analysis of the events occurring in the graft area using a division in regions provides important additional insight in course of events and local differences which would otherwise have been masked in a more global evaluation of the total graft area as a whole. The striking observation of the far more active bone formation observed at the cranial side of the biopsies in the case of SVF supplementation (7/10 biopsies) compared to the control side (1/6), as reported before [27], appears to match our current finding of higher blood vessel counts and the presence of more mature vessels (amongst others characterized by SMA+ staining) in these areas of active bone formation. The strong angiogenic potential of SVF has already been reported in numerous other studies [26,30,39]. The current findings in the six-month human model also adds to our earlier findings in previous preclinical large animal studies of our group in which we found that already after one month, SVF generates larger, more mature vessels compared to non-supplemented scaffolds [40]. The diameter of the blood vessels (mean diameter 30 µm) is well above the size of capillaries (5–10 µm) and can, therefore, be considered sufficient for bone formation [40]. Unfortunately, our study setup did not allow conclusions on blood vessel orientation, for it would have been interesting to evaluate whether the vessels would align in the principal loading direction. 

Future studies with inclusion of a higher number of patients might reveal significant differences between groups where significance could not be reached due to the relatively low number of patients included in the current study. It would be interesting to also determine the orientation of the blood vessels based on an analysis of the blood vessel cross-sectional area, to elucidate if the orientation of blood vessels may be different in the scaffold area of stem cell-treated patients compared to controls.

## 5. Conclusions

SVF, and the stem cells residing therein, ASCs, have shown high angiogenic potential, making them highly interesting for tissue regeneration in the oral and maxillofacial area, but also for other clinical disciplines. The maxillary sinus floor elevation model is unique by allowing histological examination of biopsies removed prior to dental implant placement without interfering with the clinical routine, and intra-patient treatment comparisons when using a “split-mouth” design. Within these biopsies, we were able to demonstrate an increase in the number and maturity of blood vessels, in particular in the most cranial part, when patients were treated with stem cells. Bone percentages seem to correlate with blood vessel formation and are higher in study versus control biopsies in the cranial area, in particular in β-tricalcium phosphate-treated patients. To our knowledge, this is the first study directly linking SVF-induced bone formation and blood vessel formation in a clinical setting. The pro-angiogenic, bone formation-enhancing effects of SVF provide great potential for clinical bone tissue engineering.

## Figures and Tables

**Table 1:** Patient demographics (as published earlier in [27]) showing gender and age (years), body mass index, whether the patients were treated unilaterally or bilaterally (“split-mouth design”), type of calcium phosphate bone substitute used for the maxillary sinus floor elevation procedure, and the dental implant sites according to the Fédération Dentaire Internationale (FDI) system. Selected biopsies are displayed in bold. β-TCP, β-tricalcium phosphate; BCP, biphasic calcium phosphate.

Patient Number	Gender, Age (Years)	Body Mass Index	Unilateral/Bilateral	Control/Study Side	Graft Material	Dental Implant Positions
1	♀, 58	24.5	bilateral	Control study	β-TCP	14,15,**16** 24,25,**26**
2	♀, 46	30.4	bilateral	Study control	β-TCP	14,**15**,16 24,25,**26**
3	♂, 69	35.8	bilateral	control study	β-TCP	14,15,**16** 25,26,**27**
4	♀, 59	31.2	unilateral	study	β-TCP	24,25,**26**
5	♂, 64	24.8	unilateral	study	β-TCP	15,**16**
6	♀, 57	24.2	bilateral	study control	BCP	14,15,**16** 24,**26**
7	♂, 56	28.7	bilateral	study control	BCP	15,16,**17** 25,**26**,27
8	♀, 51	30.9	unilateral	study	BCP	14,**15**,16
9	♂, 52	32.1	unilateral	study	BCP	23,25,**26**
10	♀, 51	30.3	bilateral	study control	BCP	15,**16** 25,**26**

**Table 2:** Percentages of small and large blood vessels in the scaffold area subregions (regions I, II, and III) from maxillary bone biopsies obtained from patients treated with β-TCP and BCP without stem cells (−SVF, *n* = 3) and with stem cells (+SVF, *n* = 5) by histomorphometrical and immunohistochemical analysis. β-TCP, β-tricalcium phosphate; BCP, biphasic calcium phosphate; SVF, stromal vascular fraction.

		**% of Total Blood Vessels**							
-	Size of blood vessels	Region I	Region II	Region III	Total	Region I	Region II	Region III	Total
		−SVF				+SVF			
**β-TCP**	Small	68	26	6	100	40	13	47	100
	Large	69	20	11	100	47	10	43	100
**BCP**	Small	43	37	20	100	29	43	28	100
	Large	67	18	15	100	59	7	34	100
		**% of Total CD34+ Blood Vessels**							
-	Size of blood vessels	Region I	Region II	Region III	Total	Region I	Region II	Region III	Total
		−SVF				+SVF			
**β-TCP**	Small	70	19	11	100	50	25	25	100
	Large	76	24	0	100	40	0	60	100
									
**BCP**	Small	70	14	16	100	30	51	19	100
	Large	69	0	31	100	18	42	40	100
		**% of Total SMA+ Blood Vessels**							
-	Size of blood vessels	Region I	Region II	Region III	Total	Region I	Region II	Region III	Total
		−SVF				+SVF			
**β-TCP**	Small	52	48	0	100	30	24	46	100
	Large	41	46	13	100	10	20	70	100
									
**BCP**	Small	80	0	20	100	35	43	22	100
	Large	75	0	25	100	22	19	59	100

## Supplementary Materials

The following are available online at www.mdpi.com/1996-1944/11/1/161/s1, Table S1. Percentages of small and large blood vessels from maxillary bone biopsies obtained from patients treated with β-TCP and BCP without stem cells (−SVF, *n* = 3) and with stem cells (+SVF, *n* = 5) by histomorphometrical and immunohistochemical analysis.
