# Supplementary material for: Blood Vessel Formation and Bone Regeneration Potential of the Stromal Vascular Fraction Seeded on a Calcium Phosphate Scaffold in the Human Maxillary Sinus Floor Elevation Model
Source: Materials (Basel). 2018 Jan 20;11(1):161. doi: 10.3390/ma11010161 (PMC5793659; doi:10.3390/ma11010161)
Supplement: Supplementary file 1 [file materials-11-00161-s001.pdf]

SUPPLEMENTAL TABLE

Table S1. Percentages of small and large blood vessels from maxillary bone biopsies obtained from patients treated with β-TCP and BCP without stem cells (-SVF, *n* = 3) and with stem cells (+SVF, *n* = 5) by histomorphometrical and immunohistochemical analysis. β-TCP, β-tricalcium phosphate; BCP, biphasic calcium phosphate; SVF, stromal vascular fraction; NB, native bone, TZ, transition zone; -, not present in biopsy, and therefore no % calculation possible.

		**% of total blood vessels**									
	Size of blood vessels	NB		TZ		Region I		Region II		Region III	
		-SVF	+SVF	-SVF	+SVF	-SVF	+SVF	-SVF	+SVF	-SVF	+SVF
**β-TCP**	Small	37	36	34	19	57	45	75	73	50	45
	Large	63	64	66	81	43	55	25	27	50	55
	Total	100	100	100	100	100	100	100	100	100	100
**BCP**	Small	4	0	12	25	17	21	30	60	12	17
	Large	96	100	88	75	83	79	70	40	88	83
	Total	100	100	100	100	100	100	100	100	100	100

		**% of total CD34+ blood vessels**									
	Size of blood vessels	NB		TZ		Region I		Region II		Region III	
		-SVF	+SVF	-SVF	+SVF	-SVF	+SVF	-SVF	+SVF	-SVF	+SVF
**β-TCP**	Small	45	86	65	40	81	83	74	99	100	68
	Large	55	14	35	60	19	17	26	1	0	32
	Total	100	100	100	100	100	100	100	100	100	100
**BCP**	Small	-	92	66	61	69	93	30	87	61	70
	Large	-	8	34	39	31	7	70	13	39	30
	Total	-	100	100	100	100	100	100	100	100	100

		**% of total SMA+ blood vessels**									
	Size of blood vessels	NB		TZ		Region I		Region II		Region III	
		-SVF	+SVF	-SVF	+SVF	-SVF	+SVF	-SVF	+SVF	-SVF	+SVF
**β-TCP**	Small	25	27	21	32	60	64	52	43	0	45
	Large	75	73	79	68	40	36	48	57	100	55
	Total	100	100	100	100	100	100	100	100	100	100
**BCP**	Small	33	36	26	39	65	66	-	61	50	41
	Large	67	64	74	61	35	34	-	39	50	59
	Total	100	100	100	100	100	100	-	100	100	100
